# Patient-specific seizure prediction based on heart rate variability and recurrence quantification analysis

**DOI:** 10.1371/journal.pone.0204339

**Published:** 2018-09-25

**Authors:** Lucia Billeci, Daniela Marino, Laura Insana, Giampaolo Vatti, Maurizio Varanini

**Affiliations:** 1 Institute of Clinical Physiology, National Research Council of Italy (CNR), Pisa, Italy; 2 Department of Medicine, Surgery and Neuroscience, University of Siena, Siena, Italy; University of Modena and Reggio Emilia, ITALY

## Abstract

Epilepsy is often associated with modifications in autonomic nervous system, which usually precede the onset of seizures of several minutes. Thus, there is a great interest in identifying these modifications enough time in advance to prevent a dangerous effect and to intervene. In addition, these changes can be a risk factor for epileptic patients and can increase the possibility of death. Notably autonomic changes associated to seizures are highly depended of seizure type, localization and lateralization. The aim of this study was to develop a patient-specific approach to predict seizures using electrocardiogram (ECG) features. Specifically, from the RR series, both time and frequency variables and features obtained by the recurrence quantification analysis were used. The algorithm was applied in a dataset of 15 patients with 38 different types of seizures. A feature selection step, was used to identify those features that were more significant in discriminating preictal and interictal phases. A preictal interval of 15 minutes was selected. A support vector machine (SVM) classifier was then built to classify preictal and interictal phases. First, a classifier was set up to classify preictal and interictal segments of each patient and an average sensibility of 89.06% was obtained, with a number of false positive per hour (FP/h) of 0.41. Then, in those patients who had at least 3 seizures, a double-cross-validation approach was used to predict unseen seizures on the basis of a training on previous ones. The results were quite variable according to seizure type, achieving the best performance in patients with more stereotypical seizure. The results of the proposed approach show that it is feasible to predict seizure in advance, considering patient-specific, and possible seizure specific, characteristics.

## Introduction

Epilepsy is a neurological disorder [1], characterized by the recurrence of epileptic seizures, which constitutes a nosographic entity with a considerable social impact, both due to its high incidence due to its chronicity. The lifetime prevalence of epilepsy is estimated as 7.60 per 1,000 persons while the incidence rate as 61.44 per 100,000 person-years [2].

The disorder can occur at any age, although in about 80% of the cases the crises begin before the age of 20, particularly in childhood and adolescence. In recent years there has been however an increased need of detection of the disease in elderly, both due to the increase in the average age and the increase in the incidence of cerebrovascular disorders.

In about 2/3 of the patients, seizures are controlled with drug therapy, while in the remaining cases they persist despite appropriate treatment. This is called drug-resistant epilepsy. Resistant epilepsies represent a source of serious personal, family and social distress [3].

The electroencephalogram (EEG) is widely used in the diagnosis of epilepsy, capturing the changes in neuronal activity that occur during an epileptic seizure. Such modifications, usually coincide with clinical manifestations of crisis, sometimes they can precede them for a few seconds, while in other cases they become evident only after the onset of symptoms [4]. Recently, there has been a growing interest in the analysis of the EEG signal with the aim of predicting epileptic seizures to reduce or eliminate the risks associated with it [5, 6].

It has been observed that in addition to modifications in neural activity, seizures are associated to modifications in the autonomic nervous system (ANS). Indeed, modifications of the global state of the organism, induced by modulations of the autonomic state, can induce a variation of the neuronal microenvironment and, indirectly, produce a change of the activation state of some neuronal populations [7]. One third of epilepsy patients, in addition to the alterations of the ANS often associated with the crisis (tachycardia, bradycardia, asystole, modification of ventricular repolarization), presents an altered inter-ictal electrocardiographic activity [8]. Therefore, early identification of cardio modalities could contribute to the prevention/early interruption of the crisis or, at least, to the suspension of ongoing activities in patients with treatment-resistant crises. In addition, modifications of cardiac activity could be responsible for sudden unexpected death in epilepsy (SUDEP). Such cardiac changes precede the onset of the epileptic seizure in 70% of cases, so that it has been proposed to try to modulate the heart-brain interaction for the prevention and control of focal crises in children [9].

Previous studies have reported that the analysis of electrocardiogram activity can be useful in the prediction of epileptic seizures. Some studies have analyzed Heart Rate Variability (HRV) parameters and statistically compared these indices in preictal and inter-ictal changes and found significant modifications in these features during preictal mainly indicating a higher sympathetic activity [10, 11, 12]. These changes are observed in particular 5 minutes before the seizure onset.

Other studies have used more complicated approach to evaluate the possibility of using HRV indices to predict seizure. Behabani et al. [13] used HRV indices in an adaptive decision threshold method and reached an average sensitivity of 78.59% with an intervention time of 110 Sec and false positive (FP) rate of 0.21 h^−1^. Fujiwara et al. [14] described a multivariate statistical process using time and frequency domain HRV indices and obtained a seizure prediction with a sensitivity of 91% and FP rate of 0.7 h^−1^.

Several studies have applied techniques for data classification and machine learning, in particular Support Vector Machines (SVMs) and neural networks, for the detection and prediction of epileptic seizures, using EEG data [5, 6, 15, 16]. In particular, the SVMs [17] are classification methods equipped with learning algorithms that analyze data and recognize certain patterns through the solution of non-convex optimization problems. Only one very recent study [18] presented a method for the early detection or forecasting using an SVM approach using HRV parameters. The method allowed to reveal the beginning of a seizure rom 5 min to just before the onset of a clinical/electrical seizure with a sensitivity of 94.1% and a FP rate of 0.49 h−1. This study showed the robustness of this method for seizure characterization.

It should be observed that in the aforementioned studies, the features used are quite variable, ranging from temporal indices, frequency indices or non-linear indices like those extracted from the Poincairè analysis. This is partially due to the fact that each feature, or subgroup of features, can be relevant for specific seizure onset patterns. For example, temporal lobe seizures are characterized by elevated heart rate (HR) more than the other type of seizure [19], patients with right-sided epilepsy have increased sympathetic activity (LF, low-frequency) while patients with left-sided epilepsy have increased vagal activity (HF, high-frequency) [20]; second generalized seizures are characterized by an increase in mean HR, LF/HF and SD2/SD1 ratio when compared to complex partial seizures [21]. In our study, we included a quite high number of features, selected according to the results of the previous studies, which allowed to cover all the variability present in the dataset of patients. A feature selection step, permits than to select the features which are more relevant for a specific patient.

Notably, in all the prediction studies based on ECG signals listed above, prediction is based on classical indices of HRV analyses which are extracted in the time or in the frequency domain and reflect the regulation of sympathetic and parasympathetic ANS on HR [7]. However other complementary characteristics of the cardiac signals (RR series) can be analyzed and can be useful in revealing seizure. In particular, recurrence is a fundamental property of dynamical systems, which can be exploited to characterize the system’s behavior in phase space. A powerful tool for the visualization and analysis of recurrences is the so-called recurrence plot (RP) [22]. RPs can reveal information which are not easily obtainable by other methods. The RP is the graphical representation of a binary symmetric square matrix which encodes the times when two states are in close proximity (i.e. neighbors in phase space). Based on such a recurrence matrix, a large and diverse amount of information on the dynamics of the system can be extracted and statistically quantified using recurrence quantification analysis (RQA) [23]. Importantly, no mathematical assumptions regarding the data and the generating systems are made so this tool is particularly suitable for the analysis of physiological signals which are often non-stationary [24]. RPs have been applied in the analysis and characterization of EEG signals in epileptic subjects [25, 26]. However, to the best of our knowledge, none has applied RQA for the characterization and prediction of seizures using RR series.

The aim of this study was to test present a method for the early detection or prediction of epileptic seizures based on the combination of traditional HRV parameters and RQA parameters, and the application of a SVM approach. Our approach follows a patient-specific classification method. Indeed, we hypothesized that seizure patterns across patients vary substantially and so there may be no single generic algorithm that can be applied to all patients and can achieve high sensitivity and specificity [5, 27].

## Materials and methods

### Data

The signals analyzed in this study were acquired prospectively or retrospectively from patients admitted to Unit of Neurology and Neurophysiology, Department of Neurological and Neurosensorial Sciences, University of Siena, Italy. The study was approved by the Ethical Committee of the University of Siena and performed in accordance with the Declaration of Helsinki. At the time of admission at the clinics, each patient signed a written informed consent in which agrees to the video registration and to the use of the data for a possible scientific divulgation.

The diagnosis of epilepsy and the classification of seizures was performed by two expert clinicians after a careful revision of the clinical and electrophysiological data of each patient. Specifically, the clinicians evaluated and correlated the clinical aspects of seizures (semiology, onset and history), the electrical abnormalities (Video-EEG long-term monitoring) and the neuroimaging’s findings (structural Magnetic Resonance Imaging acquired at 1.5 or 3 Tesla).

All the patients were long-term monitored with a Video-EEG, with electrodes arranged on the basis of the international 10–20 system, and with ECG. ECG was measured simultaneously with a sampling rate of 512 Hz. The onset of the seizure was identified as the earliest seizure-related change in behavior (as observed on the video recording) or EEG (a modification in the EEG typical pattern associated with an epileptic seizure as recognized by two expert epileptologists). All patients were asked to lay as much as possible in the bed in order to determine as precisely as possible the onset of the seizure from the video recording and to avoid artifacts in the EEG signals that may confuse or hide the onset of the seizure. The segments of signals in which the patients are as still as possible are then selected for the analysis. This procedure allows to reduce the influence of patient’s activity on ECG features.

Data from a total of 19 patients were acquired. These patients are drug-resistant subjects, submitted to a pre-surgical evaluation, and assuming more than one antiepileptic drug. Of these subjects 2 were excluded because they had not enough interictal periods, while other 2 subjects were excluded for poor quality signal. The final database consisted of 38 seizures from 15 patients (8 females; 7 males; age 17.6±9.9 years) mostly affected by frontotemporal epilepsy. The seizure classification was performed according to the criteria of the International League Against Epilepsy (ILAE) [28] while patients’ treatment responsivity and resistance were determined on the basis of the new ILAE 2010 criteria [29]. Table 1 gives a detailed overview of the dataset.

**Table 1:** Characteristics of the patients included in the study.

Patient ID	Age (years)	Gender	No. of Seiz.	Condition	Type of seizure	Localization	Lateralization
1	8	Male	1	Awakening	IAS	T	R
2	26	Male	1	Awakening	IAS	T	L
3	28	Female	2	Awakening	IAS	T	R
4	37	Male	2	Awakening	IAS	T	L
5	14	Female	1	Sleep	IAS	T, O	L
6	10	Female	1	Awakening	IAS	T	R
7	13	Female	8	Awakening	IAS	T	R
8	10	Female	3	Awakening	IAS	T, O	L
9	24	Male	5	Sleep (1st seizure) Awakening (other seizures)	FBTC	T	R (1st seizure) L (other seizures)
10	9	Female	2	Awakening	IAS	T	R
11	16	Male	3	Awakening	GMS	All	Both
12	9	Male	3	Awakening	FBTG	T	L (1st and 2nd seizure) Bilateral (3rd seizure)
13	34	Female	1	Awakening	IAS	T	L
14	19	Male	3	Awakening	IAS	T	L
15	7	Female	2	Sleep	IAS	T	L

IAS: focal onset impaired awareness; GMS: generalized onset motor; FBTC: focal to bilateral tonic–clonic; F: frontal; T: temporal; P: parietal; C: central; Occipital; R: right, L: left.

### ECG signals processing

**Fig 1:**
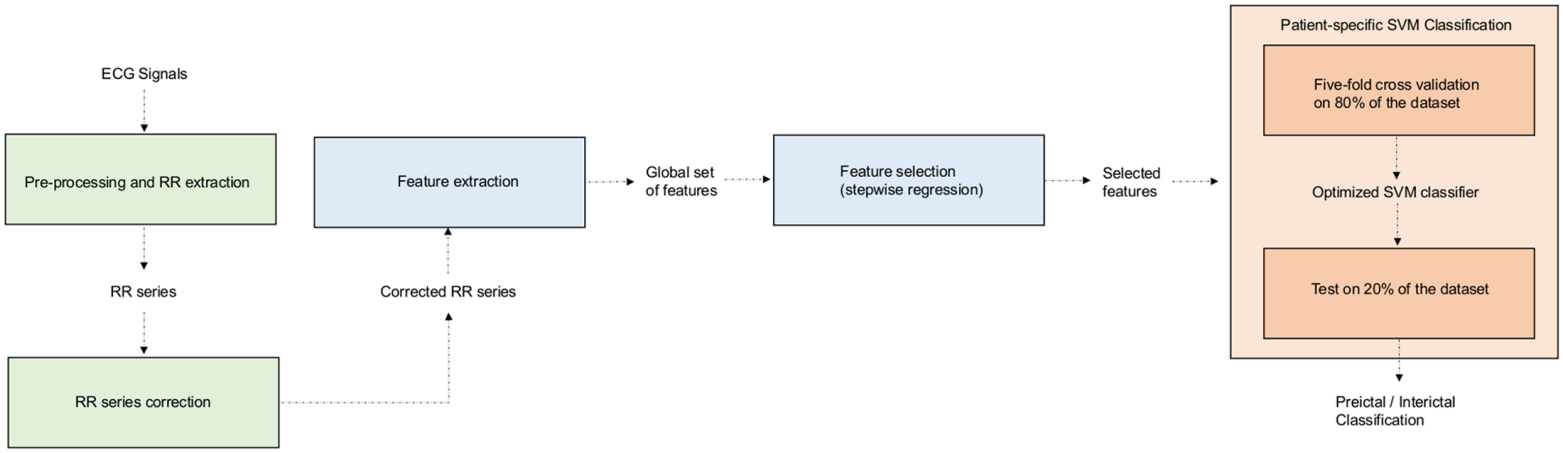
Flowchart of the proposed algorithm. The system consists of a pre-processing module, a feature extraction module, a feature selection module and finally a classification module based on Support Vector Machine. This last module includes a learning step, in which the classifier is trained, and a validation step, in which the optimized classifier is applied on the test set to classify preictal and ictal segments.

The proposed approach consists in an initial step of ECG signals preprocessing followed by feature extraction and selection. Then a classification method, based on SVM, is implemented with the aim of accurately classifying preictal and interictal segments in a patient-specific way. The flowchart of the proposed approach is represented in Fig 1. All the processing was performed using home-made Matlab scripts.

#### Pre-processing

ECG signals were first analyzed for impulsive artefacts removal, power-line interference cancelling (50Hz), baseline wandering removal, signal-to-noise ratio improvement.

For a detailed description of these pre-processing steps see [30]. Briefly, impulsive artefacts cancelling was obtained by computing the absolute difference between the original signal and the signal obtained by median filtering (60 ms window). Then if the absolute difference of a specific segment of signal exceeded a threshold (estimated considering the maximum of this difference) that segment was considered as an artefact and its values were assigned with the average of the values of the original signal immediately before and after the segment.

Baseline wandering removal was obtained by applying a low-pass first order Butterworth filter in forward and backward directions (total cutoff frequency at 3.1 Hz) and subtracting the signal obtained from original signal. The occurrence of residual artefacts, due to the filter delay in tracking fast baseline movements, was then verified. If the amplitude and the duration of residual artefacts exceed certain thresholds, a median filtering (0.26 s window) was applied.

#### RR series reconstruction

After these steps, the signal was interpolated to 1024 KHz to get a precise time location and the QRS complexes were detected to reconstruct the RR series.

For each ECG channels the QRS events were enhanced using a cascade of a (5-15Hz) bandpass filter and a derivative filter implemented as a moving average of 8 ms on the output of the comb-filter:

x(n)-x(n-k) (1)

where the delay *k* is 13 ms.

The channel with the best ECG was identified by taking into account a priori knowledge of the QRS derivative, width and pseudo-periodicity. Pseudo-periodicity was considered to discriminate ECG from noise or sporadic artefacts. In fact, QRS should occur at least once in 2 s wide windows and only few times in 0.2 s wide windows, whereas noise likely occurs in windows of both widths. On the other hand, sporadic artefacts should occur in some 8 s wide windows but only in few 2 s wide windows.

These observations and the absolute values of the derivative signal were used to build an index of quality for each ECG channel. The maximum of quality indexes was found and all the channels whose quality index was greater than 0.9 times the maximum were selected to be used in the QRS detection algorithm. The absolute derivatives of the selected channels were summed and filtered by a forward-backward Butterworth bandpass filter (6.3–16 Hz). The QRS was detected with an adaptive threshold on derivative amplitude automatically initialized and recursively updated at each new detection. Such threshold modifies with the temporal distance from the previous QRS detection, in order to avoid erroneous detection of high T wave. The fiducial point of each detected QRS was identified as the position of the maximum (minimum) of the derivative signal whose sign was determined during the initialization phase.

#### Correction of the RR interval series

**Fig 2:**
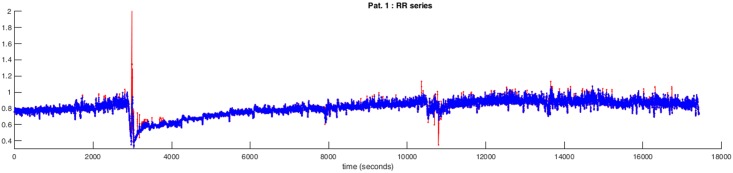
RR series before and after correction. The red line represents the series before correction and the blue line the series after the application of the correction algorithms. As an example, the RR series of Patient 1 is represented.

The autonomic nervous system controls the heart rhythm through the atrial sinus node. The RR interval series contains numerous values that do not have a sinus origin. These values can be due to heart beats of non-sinus origin (extrasystole) or may be due to errors in the recognition of the QRS complex (false positives, false negatives). Therefore, an algorithm was applied for the recognition and correction of non-sinusoidal beats to obtain a RR series that only contains variations due to the sinus node and thus reflects the activity of the ANS. The algorithm consisted in two steps:

- a finite-state algorithm based on simple rules derived from a priori knowledge and aimed at identifying and correcting outlier values. The algorithm moves, deletes or inserts QRS and their values into the RR interval series. The inserted values are equal to the ones that are expected on the bases of a priori knowledge. For example, in case of a long-lasting RR interval that is much greater than an expected value (due to a cardiac pause, an absence interval or a series of false negatives of the recognizer).
- an adaptive optimal filter in predictive form is automatically initialized on an initial range of the series. The filter uses a linear combination of previous RR values to estimate the current value. If the prediction error is less than a threshold, the current value is accepted and the filter coefficients are updated otherwise the current value is replaced with that predicted by the filter. This simple decision logic is made robust by controls and criteria such as partial updating of the coefficients and correction of the predicted in the direction of the current RR value. Fig 2 shows the RR series of Patient 1 before and after correction.

After correction, the obtained RR series was resampled at 4 Hz using a linear interpolation so that its sampling points were arranged at equal intervals.

#### Feature extraction

After pre-processing and RR series reconstruction and correction, features were extracted for each signal. Features were extracted within 180 R-R windows with 60 R-R of overlap. Notably, it has been recently demonstrated that the analysis of ultra-short-term recordings provide a reliable source of cardiac autonomic nervous system assessment [31, 32]. To reduce inter-individual variability, the whole RR was normalized with zero mean and a standard deviation of one before computing the features. A total of 20 features were computed, including time-domain features, frequency domain features and features obtained by RQA.

The time-domain features computed were the following:

- *MeanNN*: mean of RR intervals;
- *RMSSD*: root mean square of successive differences;
- *SDNN*: standard deviation of RR intervals;
- *NN50*: number of pairs of adjacent RRI whose difference is more than 50 ms;
- *PNN50*: value of *NN50* divided by the total number of N-N (R-R) intervals;
- *VAR*: variance of RR intervals;
- *SD1*: standard deviation of projection of the Poincaré plot on the line perpendicular to the line of identity that is a measure of short-term variability;
- *SD2*: standard deviation of the projection of the Poincaré plot on the line of identity that is a measure of long-term variability;
- *CSI*: Cardiac Sympathetic Index computed as:
  $$CSI=\frac{SD2}{SD1}$$ (2)
- *CSV*: Cardiac Vagal Index computed as:
  $$CSV={log}_{10}(SD1\times SD2)$$ (3)
- *CoSEn*: Coefficient of Sample Entropy, which has been specifically designed for very short RR interval time series [33, 34];
- *KFD*: Katz Fractal Dimension [35].

To compute frequency-domain features, the Generalized Short Time Fourier Transform (GSTFT) [36] was calculated.

The GSTFT is a generalization of the Short Time Fourier Transform (STFT) in which the width of the analysis window is not constant and changes with frequency according to a user defined function. Thus, the resolution in time (or the related resolution in frequency) is a general function of the frequency, which the user can define according to the signal characteristics and to the specific goal of the analysis. We have previously showed that the selection of a parametric sigmoid junction as function, gives good results in the analysis of cardiovascular time series [36].

From the GSTFT the following features were extracted:

- *LFn*: power of the low frequency band (0.04 Hz—0.15 Hz) normalized to the total power;
- *HFn*: power of the low frequency band (0.15 Hz—0.40 Hz) normalized to the total power;
- *LF/HF*: ratio of LF to HF, which is related to the sympathetic-parasympathetic balance of the ANS.

Recurrence plots (RP) proposed by Eckmann et al. [22] reveals all the times when the phase space trajectory of the dynamical system visits roughly the same area in the phase space. It is a visualization of a square matrix, in which the elements correspond to those times at which a state of a dynamical system recurs.

The RP is based on the following *N* × *N* matrix:

$$R_{i,j}=\Theta \left(\varepsilon -\|x_{i}-x_{j}\|\right)\;i,j=1,\ldots ,N$$ (4)

where *N* is the number of states of *x*_*i*_, *ɛ* is a predefined cutoff distance, ‖·‖ is the norm (e.g., the Euclidean norm) and *Θ*(·) is the Heaviside function. The cutoff distance *ɛ* defines a sphere centered at *x*_i_, if *x*_j_, falls within this sphere, i.e., the state is close to *x*_i_, then *R*_*i*,*j*_ = *1*; otherwise *R*_*i*,*j*_ = *0*. The binary values of *R*_*i*,*j*_ can be visualized in a plot with the black (1) and white (0).

The RQA is a technique for the analysis of RPs which quantifies the density of recurrence points as well as the histograms of the lengths of the diagonal and vertical lines in a recurrence plot [37]. Features extracted using the RQA were:

- *Recurrence Rate (%REC)*: quantifies the percentage of recurrent points and is calculated as
  $$\%REC=\frac{1}{N^{2}}\sum_{i,\;j=1}^{N}R_{i,\;j}$$ (5)
  The more periodic the signal dynamics, the higher the REC value.
- *Determinism (%DET)*: the ratio of recurrence points on the diagonal structures to all recurrence points, calculated as
  $$\%DET=\frac{\sum_{l=l_{min}}^{N}lP(l)}{\sum_{i,\;j}^{N}R_{i,\;j}}$$ (6)
  where *P(l)* is the number of diagonal structures whose length is *l*. *l*_*min*_ is a threshold, which excludes the diagonal lines formed by the tangential motion of a phase space trajectory. In this study *l*_*min*_ = *2*. Processes with stochastic behavior cause none or very short diagonals, whereas deterministic processes cause longer diagonals and less single, isolated recurrence points.
- *LMAX*: the length of the longest diagonal line segment in the RP, excluding the main diagonal line. The shorter the LMAX, the more chaotic (less stable) the signal.
- *Laminarity (LAM)*: the ratio of recurrence points on the vertical structures to all recurrence points, calculated as
  $$LAM=\frac{\sum_{v=v_{min}}^{N}vP(v)}{\sum_{i,\;j}^{N}R_{i,\;j}}$$ (7)
  where *P(v)* is the number of vertical structures whose length is *v*. Here, *v*_*min*_ = *2* is used. LAM decrease if the RP consists of more single recurrence points than vertical structures.
- *Trapping Time (TT)*: the average length of vertical line structures, calculated as
  $$TT=\frac{\sum_{v=v_{min}}^{N}vP(v)}{\sum_{v=v_{min}}^{N}P(v)}$$ (8)
  as for LAM, *P(v)* is the number of diagonal structures whose length is *v* and *v*_*min*_ = *2*. TT estimates the mean time at which the system will abide at a specific state or how long the state will be trapped.
- *Entropy (ENT)*: the Shannon entropy of the frequency distribution of the diagonal line lengths, calculated as
  $$ENT=-\sum_{l=l_{min}}^{N}P(l)\text{ln}P(l)$$ (9)
  where *P*(*l*) is the probability density of the diagonal structure whose length is *l* and it is defined as *P*(*l*)/sum(*P*(*l*)). ENT is considered as a complexity measure of the system: the more complex the deterministic structure, the larger ENT value.

### Segmentation of feature vectors

Following the feature extraction phase, each vector of features was segmented in the following states:

- *Ictal*: from the seizure onset to the end of seizures, as marked by the clinicians. This segment is of varying length but is typically close to 3 minutes long;
- *Preictal*: from 15 minutes prior to the seizure onset to the seizure onset;
- *Interictal*: non-seizure data preceding the preictal state or proceeding the postictal state which are apart from at least 50 minutes from seizure [14].

### Feature selection

Before applying classification, a feature selection step was included. Different feature selection algorithms were tested namely the neighborhood component analysis, the neighborhood component analysis for regression, the stepwise regression analysis and the ReliefF algorithm. In our sample, the stepwise regression analysis gave the most accurate results.

The stepwise regression analysis works by adding and removing terms from a multilinear model according to their statistical significance in a regression. The method begins with an initial model and then compares the explanatory power of incrementally larger and smaller models. At each step, the *p* value of an *F*-statistic is computed to test models with and without a potential term. If a term is not currently in the model, the null hypothesis is that the term would have a zero coefficient if added to the model. If there is sufficient evidence to reject the null hypothesis, the term is added to the model. Conversely, if a term is currently in the model, the null hypothesis is that the term has a zero coefficient. If there is insufficient evidence to reject the null hypothesis, the term is removed from the model. The method proceeds as follows:

1. Fit the initial model.
2. If any terms not in the model have *p*-values less than an entrance tolerance, add the one with the smallest *p* value and repeat this step; otherwise, go to step 3.
3. If any terms in the model have *p*-values greater than an exit tolerance, remove the one with the largest *p* value and go to step 2; otherwise, end.

The method terminates when no single step improves the model.

### Classification

Our patient-specific approach to seizure prediction is based on binary classification of ECG segments using a machine learning algorithm. The aim is to classify ECG segments as either preictal or interictal based on the selected features. Thus, the vectors of selected features were submitted to an SVM for classification. This method, developed by Vapnik [38], is a powerful tool often used for solving supervised classification problems due to its generalization ability. An SVM classifiers works by maximizing the margin between the training data and the decision boundary. The boundary is a hyperplane that optimally separate the classes. The subset of patterns those are closest to the decision boundary are called *support vectors*.

Let *{****x***_*i*_,*y*_*i*_*}* with *i = 1…N* be the training data with $x_{i}\in R^{d}$ the input vector containing the selected features, and *y*_*i*_ *∈ {− 1*, *+1}* the classes. In the case of a linearly separable classification problem, the model constructs a hyperplane as follows:

$${(w}^{T}\phi (x)+b)=0$$ (10)

were the function *ϕ(****x****)* maps the vector of features ***x*** to a high dimensional space $R^{D},\;w\in R^{D}$ is weight vector and *b* is scalar bias.

The output of the classifier can be determined by the following equation:

$$f(x)={sign\;(w}^{T}\phi (x)+b)$$ (11)

In real applications classes are not linearly separable i.e. data of classes are overlapping and misclassifications should be allowed. Thus, the unknown variables ***w*** and *b* can be found by solving the following optimization problem:

$${min}_{w,\;b,E}\{\frac{1}{2}(w^{T}w)+C\sum_{i=1}^{l}E_{i}\}$$ (12)

such that:

$$y_{i}{(w}^{T}\phi (x)+b)\geq 1-E_{i},\;E_{i}\geq 0,\;i=1,\;\ldots ,\;N$$ (13)

With *C > 0*, (penalty parameter of the error term) is a regularization factor which determines the trade-off between model complexity and misclassification error.

The kernel function is given by the relation:

$$k(x,x_{i})={\phi (x)}^{T}\phi (x_{i})$$ (14)

In this paper, a radial basis function (RBF) was used as the kernel function. The RBF kernel is defined as follows:

$$k(x,x_{i})=e^{-{\gamma \|x-x_{i}\|}^{2}},\;\gamma >0$$ (15)

where ***x***_*i*_ is the input sample, ***x*** is the space of the training set, and ‖·‖ represents the Euclidean distance operator. The performance of the SVM classifier depend by two parameters: the penalty parameter (*C*), which controls model overfitting, and the parameter gamma (*γ*), which controls the model’s degree of nonlinearity. To obtain the optimal classification performance, it is important to find the best combination of these parameters. In this work, an automatic optimization function in Matlab was used, which find hyperparameters that minimize five-fold cross-validation loss.

Cost-sensitive SVMs (CSVMs) was used due to the fact that the datasets are unbalanced [39]: the number of interictal segments are much greater than the number of preictal ones.

For each dataset D, the weight values used for interictal and preictal classes were respectively set at 1 and inter_D_/pre_D_ where inter_D_ is the number of interictal segments and pre_D_ is the number of preictal segments [6].

In a first experiment we applied a cross-validation to all patients with the aim of classifying preictal and interictal segments. For each patient, we randomly selected 80% of the dataset (set of the vectors of feature calculated in each time window) and evaluated the prediction rate by testing the model on 20% of the dataset that was reserved for testing. Due to the low number of preictal segments, the cross validation of the SVM classifier was performed on the whole dataset deserved for training. A five cross-validation training was performed. The optimal SVM classifier was selected as the one with the lower classification loss, i.e. the one with the lower predictive inaccuracy. The performance of this classifier was tested on the test set and the performance were calculated.

In a second experiment, we applied a double cross-validation just in those patients who had at least 3 seizures (5 out of 15 patients in the dataset) with the aim to predict a seizure block (preictal + interictal) on the basis of the other ones. According to double cross-validation method, data were partitioned into two subsets: training set and test set. The training set was separated into subdivisions: learning set and validation set. The double cross-validation method allows to have an unbiased estimation of SVM accuracy [40]. A similar approach was previously applied in the prediction of seizures using EEG recordings [5, 41].

In a patient with N seizures and I-hour-long interictal recordings (at least 50 minutes far from the seizure), we separated the dataset into N block, each containing (I/N)-hour of interictal recording and 15 min of preictal. Then, we used the last seizure block for testing set and the other (N − 1) blocks for training set. To consider reality and physiological condition, classifier was trained on earlier and later seizures were used for testing [41].

To establish an optimal SVM classifier in training, we perform fivefold cross-validation by randomly selecting 80% of the training set (learning set) and validating the model on the remaining 20% of the training set (validation set) to check if the model is well-fit. Once the SVM model was trained, the performance e was evaluated by testing the model on the fold that was reserved for testing. Since we want at least two seizures blocks for training, the seizure blocks that were predicted were from the third to the last one. Thus, this process is repeated (N-2) times and the average prediction rate is reported. The prediction time was also calculated as the time interval from the first vector of feature that was classified as “pre-seizure” and the seizure onset.

### Performance evaluation

For each patient, the results were assessed in terms of sensitivity, specificity, accuracy and false prediction rate per hour (FP/h), Sensitivity (Sens) and specificity (Spec), which reflect performance of imbalanced classification, are defined as following [39]:

$$Sens\%=\frac{TP}{\left(TP+FN\right)}\times 100$$ (16)

$$Spec\%=\frac{TN}{\left(TN+FP\right)}\times 100$$ (17)

Accuracy (Acc) was calculated as:

$$Acc\%=\frac{TP+TN}{TP+TN+FP+FN}\times 100$$ (18)

In addition, FP/h was calculated as the number of FP divided by the number of available hours of interictal.

TP, TN, FP, and FN were defined as follows:

- TP: correctly classified preictal segments;
- TN: correctly classified interictal segments;
- FP: incorrectly classified interictal segments;
- FN: incorrectly classified preictal segments.

## Results

Different features were selected for each patient in the feature selection step. From 3 to a maximum of 13 features were selected with an average of 6.7 features per patient. Table 2 shows the features selected for each patient. In the last row of the table the number of patient for which each feature is selected are reported. The most frequently (half number of patients or more) selected features were: meanNN, pNN50, CosEn, LAM, HF and LF/HF.

**Table 2:** Selected features for each patient. In the last row of the table were the number of patient for which each feature is selected is reported, the more frequently selected features are highlighted in bold.

Pat. No.	MeanNN	SDNN	RMSSD	pNN50	VAR	SD1	SD2	CSI	CSV	KFD	CoSEn	%REC	DET	LMAX	ENT	LAM	TT	LF	HF	LF/HF
1	x				x						x				x	x		x		x
2	x	x	x	x			x	x	x	x	x			x			x		x	x
3									x		x	x				x	x		x	
4	x		x							x		x		x						x
5	x	x		x	x		x				x	x								
6	x				x			x						x		x	x	x		
7				x						x	x			x						x
8	x			x	x	x				x	x	x							x	x
9	x			x						x							x		x	
10				x	x			x			x					x		x	x	
11								x			x								x	
12				x				x				x				x				x
13												x	x	x		x				x
14	x			x		x				x	x		x	x		x		x	x	
15	x			x							x					x				x
TOT	**9**	1	2	**9**	6	1	2	5	2	5	**9**	6	2	5	1	**8**	4	3	**7**	**8**

**Fig 3:**
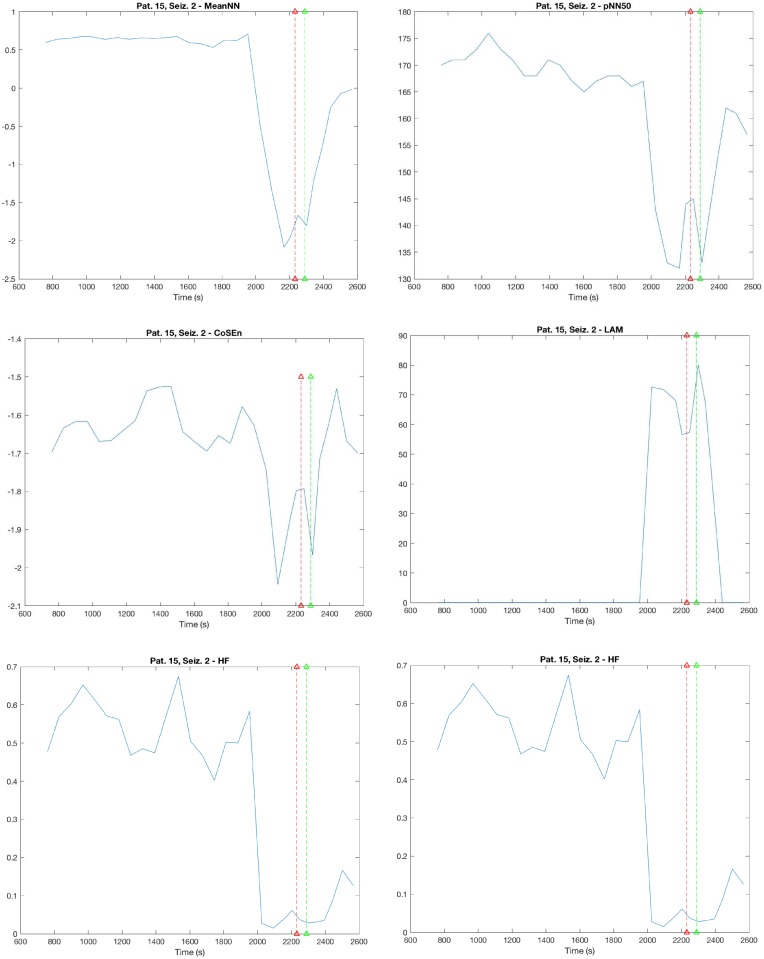
Dynamics of the most discriminant features. The image represents the dynamic of the features with the most discriminant power for preictal and interictal segments according to the stepwise regression analysis. Each panel presents dynamic changes in one particular HRV parameter from 25 minutes before seizure onset to 5 minutes after the end of the seizure. Seizure onset and seizure end are represented by the vertical red and green lines respectively. An exemplificative seizure from one patient is reported (Patient 15, Seizure 2). a) meanNN, b) pNN50, c) CosEn: coefficient of sample entropy, d) LAM: laminarity, e) HF: high frequency, f) LF/HF: ratio between low and high frequency.

Fig 3 shows the dynamics of the most frequently selected features for one seizure in Patient 15. The signals are displayed from 25 minutes before seizure onset to 5 minutes after the end of the seizure. It can be observed that the meanNN, pNN50, CoSEn and HF decrease from preictal to the ictal phase while LAM and LF/HF increase.

**Fig 4:**
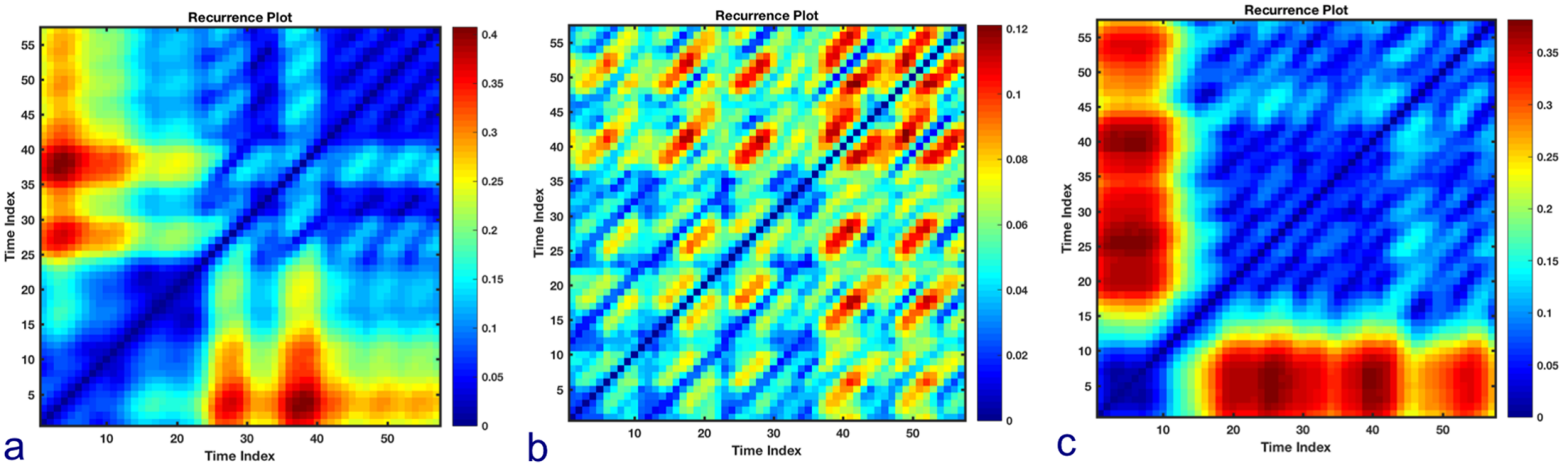
Trend of recurrence plots. Examples of recurrence plots (RPs) of RR intervals’ phase space trajectory for Patient 15, Seizure 2. (a) RP during the interictal phase (60 minutes from the seizure); (b) RP during preictal phase and (c) RP during postictal phase. It can be observed that the RP of the preictal phase presents more regular patterns and long diagonal lines while the RPs of the interictal a more disorganized pattern, with shorter diagonal lines, hence lower determinism. In the RPs the distance to the next recurrence point is color-coded.

The temporal trends of the recurrence plots showed a modification of the appearance during the progression of seizure becoming more regular and ordered in the preictal phases compared to interictal and postictal phases (Fig 4).

The classification results on the test epochs by the trained-SVM with the selected features for each patient are reported in Table 3. The average Acc, Sens%, Spec% and FP/h were 88.06%, 89.06% 89.34% and 0.41, respectively.

**Table 3:** Classification results for experiment 1.

Pat. ID	No. of Seiz.	Interictal hours	Accuracy	Sens%	Spec%	FP/h
1	1	3.09	91.49	100.00	90.70	0.36
2	1	2.61	97.56	100.00	97.37	0.1
3	2	6.45	79.17	100.00	77.78	0.87
4	2	8.35	94.33	100.00	93.94	0.24
5	1	2.91	91.30	70.00	93.02	0.28
6	1	1.26	100.00	100.00	100.00	0.00
7	8	3.81	82.35	70.00	92.45	0.29
8	3	2.38	87.50	88.57	84.62	0.45
9	5	4.53	89.58	65.22	97.26	0.11
10	2	2.34	75.00	75.00	75.00	0.99
11	3	4.40	83.33	83.33	83.33	0.66
12	3	3.69	92.41	100.00	90.00	1.12
13	1	1.11	90.00	100.00	88.24	0.48
14	3	4.07	91.43	90.83	91.67	0.38
15	2	2.36	88.46	100.00	85.00	0.57
Average	2.67	3.56	88.86	89.06	89.34	0.41

In the second experiment, we applied a double cross-validation to predict unseen seizures just in those patients who had at least 3 seizures. The results for these six patients are reported in Table 4. For each patient the performance for each seizure and the average performance are reported. The mean performance on all seizures and all patients are Acc: 74.62%, Sens: 70.08%, Spec: 76.98% and FP/h: 3.36.

**Table 4:** Classification results for experiment 2.

Pat. ID	No. of Seiz.	Accuracy	Sens%	Spec%	FP/h	Prediction time (min)
7	8 Seiz. 3 Seiz. 4 Seiz. 5 Seiz. 6 Seiz. 7 Seiz. 8 Average	85.42 70.00 56.36 84.75 88.19 98.21 80.48	53.33 11.00 95.45 76.92 100.00 95.65 72.06	100.00 100.00 30.30 90.91 78.79 100.00 83.33	0.00 0.00 14.00 1.83 4.28 0.00 3.35	9 15 15 15 15 15 14
8	3 Seiz. 3	75.31	81.45	70.00	3.50	15
9	5 Seiz. 3 Seiz. 4 Seiz. 5 Average	58.00 89.90 86.60 88.25	34.00 84.62 62.5 60.37	64.38 91.78 94.52 83.56	5.00 1.00 1.00 2.30	15 13 15 14.3
11	6 Seiz. 3 Seiz. 4 Seiz. 5 Seiz. 6 Average	85.00 67.00 60.00 43.00 63.75	54.00 67.00 100.00 83.33 76.08	100.0 67.00 41.00 37.00 61.25	0.00 2.00 3.50 3.80 2.35	13 15 15 15 14.5
12	1 Seiz.3	62.00	58.00	65.71	6.0	15
14	3 Seiz. 3	88.03	73.00	98.02	1.50	10
Average		74.62	70.16	76.98	3.40	13.7

## Discussion

In this study, we proposed a method to predict seizures on the basis of ECG in a patient- specific way. We felt that a patient-specific approach is more suitable to characterize and predict seizure since the autonomic changes in epilepsy can be largely dependent on several factors including the localization of seizure, lateralization and drugs [20].

Two important aspects of our study were that we applied a patient-specific approach to classify preictal and interictal segments and that we evaluated the ability to predict unseen seizure on the basis of the previous ones. Notably, our dataset has quite a large variability including different type of seizures with a different region and hemisphere of onset, different conditions (sleep or awakeness) and different patient’s age. Such set allowed to evaluate how robust is the algorithms in predicting seizure of different kind and recorded in different conditions. Previous research has shown that the autonomic modifications in response to seizure are different in children and adolescents compared to adults. In particular, it has been observed that children with refractory epilepsy have lower HRV resulting from parasympathetic tonus reduction, suggesting that the decreased HRV in children occurs via different mechanisms than in adults [42, 43]. Moreover, it has been observed that tachycardia occurs in 98% of children suffering complex partial seizures of temporal lobe origin, more frequently than in adults [44]. Besides the influence of age, the localization of seizures can also influence the autonomic response. Previous studies reported that secondary generalized and complex partial subjects manifest significantly different autonomic behaviors [10]. Lateralization of seizures can also influence autonomic state with right-sided epilepsy characterized by increased sympathetic activity (tachycardia and LF) while left-sided epilepsy determined an increased vagal activity (bradycardia and HF) [9]. In addition, the autonomic response is very different between awake and the sleeping state, which has unique, stage-specific autonomic heart-rate modulation [13, 45]. In this regard, it should be noticed that in most of the patients (except for Patient 9) all the seizures are recorded during sleep or during awake state so that the condition in which the seizures are registered is homogeneous for the single patient. This allows to have a more robust prediction for each single patient. Given these considerations, we expected that, the patient-specific methodology could have been more successful for some patients than others and in predicting some seizure than others; in particular, in those patients who have repeatable and stereotypical seizures we expected the algorithm would have been more efficient.

In a first experiment, we applied a cross-validation SVM approach in all the 15 patients and we obtained a good mean accuracy of 88.86% and a good mean sensitivity of 89.06% with a low number of FP/h (0.41). This result suggests that seizures could be accurately predicted using the proposed patient-specific approach.

In a second experiment, we adopted a double-cross validation approach to predict seizure on the basis of previous ones. This approach was previously applied only for predicting seizures with EEG signals [5, 41]. Using this approach, we used in-sample optimization and assessed the results with out-of-sample testing (a test set is never involved in training corresponding to unseen seizures). Thus, we could have obtained lower sensitivity and/or specificity than algorithms trained and tested on the exact same datasets; but in this way the results are much more stable [40].

According to our expectations, seizure predictions was quite dependent on the type of seizures and/or condition and thus the algorithms worked better for some patients than others. Indeed, in both the two experiments sensitivity was quite low for Patient 9 in which seizures started in the right hemispheres and then moved to the left one and the seizures are registered both during sleep and awake state and for Patient 5, in which seizure onset is localized in several regions of the brain rather than be focal. When applying a double cross-validation to predict unseen seizures while training the model on the previous ones, we confirmed that for Patient 9 the performance of the algorithm was quite poor probably because of the changes in condition (from sleep to awakeness) and lateralization (from right to left).

The average prediction time was quite high, 13.7 min, meaning that a long period is available to trigger an intervention or to secure the patient before the upcoming seizure.

Literature regarding prediction algorithms using ECG signals is quite poor. Kerem and Geva [45] proposed an unsupervised fuzzy clustering algorithm to predict partial seizures with temporal-lobe localization. The method was applied in a quite small dataset consisting in 21 seizures collected in 8 patients both during sleep and awakeness and achieved a sensitivity of 86%. The seizure prediction window was 1.5–11 min. More recently, Behbahani et al. [13] employed an adaptive decision threshold method for raising alarms and for predicting seizures: predictions were made when selected features exceeded the decision thresholds. The dataset used for this study was quite large as it consisted in a total of 170 seizures collected in 16 patients both during sleep and awakeness. The method showed an average sensitivity of 78.59%, and an average false prediction rate of 0.21/hr with a prediction window of about 5 min. In another study [14], seizure prediction was obtained by applying multivariate statistical process control (MSPC) to eight HRV features. The application results of the proposed method obtained a sensitivity of 91%, and a false positive rate of about 0.7/hr with a prediction window of 15 min. However, the dataset was quite small as it consisted in 11 awakening seizures from 8 patients.

A very recent study applied SVM algorithm to classify preictal and interictal segments on the basis of HRV features [18]. The sample size was quite similar to our (34 seizures from 12 patients). In this study, seizures were predicted from 5 min to just before the onset of a clinical/electrical seizure with a sensitivity of 94.1% and a FP rate of 0.49/hr.

In our study, we obtained a sensitivity of 89.06% when classifying seizures from single subject which was higher than that obtained in [18] and [45]. Fujiwara et al. [14] obtained a slightly higher sensitivity but the FP rate was a bit greater than our and the dataset was smaller. The highest sensitivity was that obtained by Pavei et al. [18]. However, it is important to notice that this performance refers to the ability of correctly classifying preictal segments of the whole database and no information about the performance on each subject (or each seizure) is provided.

Another important point is that we were able to correctly classifying preictal segments from 15 min to seizure onset. A 10–20 min timeframe for seizure prediction is in accord with a transition to a low-dimensional, non-linear dynamics state in intracranially recorded electrical activity [46], as well as with a significant and sustained increase in blood flow measured by SPECT [47] and by subdural thermal diffusion flowmetry sensors [48]. Some studies wed a rather short preictal segment of 5 minutes [9, 18, 49]. This temporal restriction excludes the possibility, supported by previous studies, that changes in autonomic activity occurs much earlier [50].

As regards the more relevant features that we identified in the classification of seizure prediction, meanNN, pNN50, CosEn, LAM, HF and LF/HF resulted as the most frequently selected by the stepwise regression analysis. This finding, partially reflect the results of our previous study aimed at assessing the significant differences in ANS among the preictal, the interictal and ictal phases [51].

Previous studies have reported that heart rate changes preceded the seizure onset on EEG in 70% of the cases [9]. Limbic structures are responsible of these changes which are more prominent when the volume of cerebral structures recruited into a seizure is increased [52]. In particular, according to previous studies, tachycardia occurs in 86.9% of all seizures, whereas bradycardia is documented only in 1.4%. Localization of seizures influences this pattern with HR increase being more pronounced in patients with mesial temporal lobe epilepsy (TLE) as compared with those with non-lesional TLE or extratemporal epilepsy [53]. Consistently, we observed a decrease of meanNN (increase of HR) for example in Patient 15 (Fig 3).

We also observed a role of pNN50 in predicting seizures. In particular, in we observed a decrease in NN50 in patients with TLE (Fig 3), suggesting a deterioration of HRV which could be an indication of increased cardiovascular risk, including mortality [54]. Significantly decreased in NN50 was previously reported during epilepsy [55]. In addition, it was recently observed, using a K-nearest neighbors (KNN) classifier, that NN50 and PNN50 were the most relevant features for predicting epileptic seizures [56].

We also observed an involvement of HF and LF/HF, specifically with a decrease in HF and an increase of LF/HF (Fig 3). Literature shows that epileptic seizures affect the autonomic nervous system and consequently activities of both sympathetic and parasympathetic nerves. Most of the studies reported a decrease in HF, corresponding to a decrease in vagal activity, sometimes associated with an increase LF, mainly related to sympathetic activity [57]. The decrease in vagal activity (and possibly the increased in sympathetic activity) during the preictal phase, associated to the tachycardia and/or tachyarrhythmia usually observed, could mean a difficulty in vagal activity in restoring normal heart rhythm and thus favor the development of arrhythmias. This mechanism could be associated to the increased risk of SUDEP associated to epilepsy [57].

Importantly, we observed also a significance of RQA parameters in preictal phase, that were for the first time applied for the characterization of ANS during seizures. In particular, LAM was the most frequently selected for classification but also %REC and LMAX were often selected. These parameters usually increased in preictal state to the ictal state (Fig 3). These changes, previously observed applying RQA analysis to EEG signals in epilepsy [58, 59], suggest an increase in synchronization associated to seizures and could reflect the synchronization of neurons during seizures. Consistently CosEn is one of the most frequently selected features. Entropy estimators measure the degree of regularity of a signal by counting how many template patterns repeat themselves, thus repeated patterns imply order [33]. Previous studies have observed, marked differences in entropy between ictal and interictal periods [7, 60]. The decrease in CosEn during preictal and ictal states indicate that the time series has a predictable pattern with minimal randomness.

Overall the findings of this study suggest significant changes in ANS at least 15 min before seizures. Thus, the proposed algorithm could help in the prediction of the seizures enough time in advance to prevent adverse effects and to intervene. According to the variability in the classification performance for the different patients or seizure, we conclude that quite a large between-patient and within-patient variability exists in the autonomic response associated to seizures and thus a patient-tailored or even seizure-specific approach, rather than a universal system and features, should be adopted and realized for individualized alarm system.

Further studies are needed to confirm these preliminary results in a larger number of subjects addressing some of the limitations of the present study. First, our dataset was too small and heterogenous (in terms of age, gender, seizure location) to allow a meaningful subgroup analysis. In a larger sample, it would be possible to apply the SVM classification in different homogeneous subgroups to evaluate how the performance change according to the different characteristics of the subjects including seizure location, lateralization and patient condition (sleep or awakening). In, addition, we did not control for possible confounding factors such as antiepileptic drugs. We know that antiepileptic drugs, have an influence on measured ECG features [57]. However, in this study, due to the pharmacological variety of the different patients, the fact that most of them are in polytherapy and finally considering that there is an extreme variability in the response to the various drugs for the different patients, it was almost impossible to assess the role of the different drugs as confounding factor. This limitation, is partially addressed by using a patient-specific approach, so that the method for classification is tailored individually. In a larger sample, it could be possible to assess how the classification performance change in different subgroups with a homogeneous therapy. In the future it will be also important to combine in this patient-specific classification approach ECG and EEG data to obtained a more sensible and specific prediction of different kind of seizure. Indeed, previous research have showed that the combination of these signals improves the performance of prediction algorithms [61, 62].
